# Delivery of chlamydia screening to young women requesting emergency hormonal contraception at pharmacies in Manchester, UK: a prospective study

**DOI:** 10.1186/1472-6874-9-7

**Published:** 2009-03-26

**Authors:** Loretta Brabin, Grace Thomas, Mark Hopkins, Karen O'Brien, Stephen A Roberts

**Affiliations:** 1 Academic Unit of Obstetrics & Gynaecology, University of Manchester, Manchester, M13 OJH, UK; 2 Liverpool Specialist Virology Department, Royal Liverpool University Hospital, Liverpool, L7 8XP, UK; 3 Commissioning Directorate, Manchester Primary Care Trust, Manchester, M21 7RL, UK; 4 Health Methodology Research Group, University of Manchester, Manchester, M13 9PL, UK

## Abstract

**Background:**

More women are requesting Emergency Hormonal Contraception (EHC) at pharmacies where screening for *Chlamydia trachomatis *is not routinely offered. The objective of this study was to assess the uptake of free postal chlamydia screening by women under 25 years who requested EHC at pharmacies in Manchester, UK.

**Methods:**

Six Primary Care Trusts (PCTs) that had contracted with pharmacies to provide free EHC, requested the largest EHC providers (≥ 40 doses annually) to also offer these clients a coded chlamydia home testing kit. Pharmacies kept records of the ages and numbers of women who accepted or refused chlamydia kits. Women sent urine samples directly to the laboratory for testing and positive cases were notified. Audit data on EHC coverage was obtained from PCTs to assess the proportion of clients eligible for screening and to verify the uptake rate.

**Results:**

33 pharmacies participated. Audit data for 131 pharmacy months indicated that only 24.8% (675/2718) of women provided EHC were also offered chlamydia screening. Based on tracking forms provided by pharmacies for the whole of the study, 1348/2904 EHC clients (46.4%) who had been offered screening accepted a screening kit. 264 (17.6%) of those who accepted a kit returned a sample, of whom 24 (9.1%) were chlamydia-positive. There was an increase in chlamydia positivity with age (OR: 1.2 per year; 1.04 to 1.44; p = 0.015).

**Conclusion:**

Chlamydia screening for EHC pharmacy clients is warranted but failure of pharmacists to target all EHC clients represented a missed opportunity for treating a well defined high-risk group.

## Background

The National Chlamydia Screening Programme (NCSP) is an opportunistic programme that offers screening to sexually active men and women under the age of 25 in clinical and non-clinical settings [1]. Prevalence of chlamydia is highest in this young age group and more infections are detected in healthcare settings than in the general population [2]. Active case finding for genital chlamydia infection through screening of "at risk" populations can reduce the prevalence of infection if coverage is sufficiently high [3]. In pilot studies conducted prior to the introduction of the NCSP in 2003, screening was taken up by about 50% of sexually active women aged 16–24 years [4], a level not achieved to date by the NCSP [1]. For transmission to fall, the annual screening and testing rate of sexually active young adults under 25 in a variety of healthcare and non-healthcare settings (including tests at genitourinary medicine clinics) must be sustained above 35%, together with effective partner notification and management [5,6]. The success of opportunistic screening depends on users accessing services and adequate levels of awareness and training of health providers [7]. Some researchers have argued that more systematic approaches for calling and recalling the target population would have a higher impact on prevention of chlamydia-associated morbidity than opportunistic screening [8]. Using general practitioner registers to invite young men and women to return home-collected samples [9] however, had a lower uptake than expected. The most effective approaches for screening women for whom coverage is low have yet to be determined [10].

Meanwhile opportunistic screening at pharmacies has been encouraged by the NCSP. Approximately 11% of women and 14% of men accessed chlamydia screening at pharmacies in 2006/07 [1] and a scheme was launched by the Department of Health to assess uptake of free chlamydia screening for both young men and women through Boots pharmacies in 31 London Primary Care Trusts (PCTs) [11]. Pharmacies have been increasing their range of services and the government recognises them as a mainstream contributor to primary care and public health [12]. Since 2000 EHC has become increasingly available from pharmacies in a number of European countries, Canada, New Zealand and Australia [13]. EHC is indicated to prevent pregnancy after unprotected sexual intercourse, which also exposes women to the risk of sexually transmitted infections (STIs) [14-16]. In the UK, reclassification of EHC from a "prescription only" to an "over the counter" product in 2001 made EHC available on request. This shift increased early access after unprotected sex, was generally well accepted by the industry, and was well-rated by women [17] although it had little impact on unwanted pregnancy rates [18-20]. Within three years, 27% of EHC requests were handled through pharmacies [21]. This shift in EHC provision away from sexual health clinics increases the likelihood that this sub-group of high risk women will not be screened for chlamydia. As such, a targeted chlamydia case-finding approach among EHC pharmacy clients might be appropriate. Pharmacists involved in EHC schemes are trained to assess Fraser competency of a minor (<16 years) to give informed consent to medical treatment, and to advise on the risks of STIs resulting from unprotected sex [20]. A pilot study in three pharmacies in London, in which testing and treatment were offered on site, suggested that chlamydia screening was acceptable to pharmacists [22]. The purpose of our study was to assess targeted delivery of free postal chlamydia screening to women requesting EHC. Postal screening was offered as pharmacies could offer this with little inconvenience, and it would not require them to have on-site facilities for sample collection.

## Methods

The North West Multi Centre Research Ethics Committee approved the study. The NCSP (which was locally known as RUCLear) was rolled out gradually across England and was not implemented in Manchester until December 2006. This allowed the study to be completed from January to December 2006 during the planning stages for its introduction and in consultation with the RUClear co-ordinator.

### Selection of pharmacies

Patient group directions (PGD) constitute a legal framework which allows certain health professionals to supply medicines to groups of patients that fit the criteria laid out in the PGD. PCTs are responsible for local pharmaceutical services, and six PCTs in Manchester had contracted with 76 pharmacies to provide free EHC (progestogen-only Levonelle-2) under a PGD to women under 25 [23]. The PCTs requested pharmacies to also offer postal chlamydia screening, although no additional remuneration was provided. A pragmatic decision was taken only to include pharmacies that supplied at least 40 women with EHC per year.

### Procedures

Accredited EHC pharmacists were given additional training on chlamydia screening during EHC refresher courses or individually by a sexual health research nurse (GT). Pharmacists were instructed to offer the postal kit after completing the EHC protocol when advising on STI risks, and to explain the purpose and contents of the screening kit. Testing kits were discretely packaged and contained an information sheet, a consent form, a brief pre-piloted questionnaire on symptoms, previous EHC use and chlamydia treatment and demographic characteristics, as well as instructions on taking and returning the sample to the laboratory in the pre-paid envelope. Women were advised to return the urine sample within two weeks. Urines, rather than self taken swabs, were collected in line with NCSP standard practice. Details of local sexual health services were also provided. Each kit had a unique number and central records were kept, indicating to which pharmacy the kits had been issued and which kits were returned. Pharmacies were requested to keep records of the numbers and age group of each EHC client, whether she accepted or declined the screening offer, and the kit number if accepted. These details were faxed to the research nurse every month and she regularly visited all pharmacies to ensure kits were available and to monitor data recording. A face-to-face structured exit questionnaire was conducted with all pharmacists who continued to the end of the study and had not moved outside the area.

### Laboratory tests and treatment

Urines, consent forms and questionnaires were returned together to the Liverpool Specialist Virology Centre and screened for the presence of *Chlamydia trachomatis *using the Roche COBAS Taqman CT test (Roche Diagnostics Ltd, Burgess Hill, UK) as described by the manufacturer. All specimens identified as inhibitory or *C. trachomatis *positive were retested using the Artus *C. trachomatis *Plus PCR Kit after DNA isolation using a Qiagen M48 Biorobot and Magattract Virus Mini M48 Kit (Qiagen Ltd, Crawley, UK), both according to the manufacturer's instructions. Questionnaires and consent forms were collected weekly from the University of Liverpool but women were who tested chlamydia-positive or were required to give another sample were notified immediately. Participants could opt for treatment by the research nurse at Manchester Centre for Sexual Health or a family planning clinic. If the client chose to book her own appointment, the research nurse contacted the client to ensure she had attended and received treatment and had been advised about treatment of partners. Two participants were not treated because they gave incorrect details and/or their GP (if they had consented for GP contact) could not be traced.

### Data analysis

It should have been possible to estimate the number of eligible women treated from the tracking forms that pharmacists had agreed to complete for all EHC clients but 17% of samples sent to the laboratory were not recorded on these forms. Hence the number of women who were offered EHC, who should have been given a screening kit, was estimated from routine audit data collected by the PCTs for the EHC programme. Due to PCT organisational changes, not all the routine data could be accessed. The denominator for Analysis (1), to determine the number of women supplied with EHC who had been offered screening, was therefore based on an audit of those periods for which the data were recorded by the PCTs. Analysis (2) uses the denominator provided from the tracking forms to determine the proportion of women who the pharmacist recorded as accepting the screening kit. The number of accepted kits is the denominator for analysis (3) which describes the proportion of kits returned to the laboratory. Binomial 95% confidence intervals were computed for chlamydia prevalence. Where age data were grouped, trends in age were assessed using logistic regression with age group as the covariate. Logistic regression against calendar age was used where individual data were available.

## Results

There were 35 eligible pharmacies, and 33 participated in the study. The types of pharmacy represented were 18 large chains (ie branch stores of large corporations), 12 independents, and 3 small chains. 15 were located in shopping precincts or residential areas, 4 were attached to surgeries and one was in a superstore. The majority (20) had only one pharmacist. Six pharmacies interrupted or ceased participation in both EHC provision and chlamydia screening because the EHC-trained pharmacist moved and was not replaced. One pharmacy withdrew early from the study after its own brand of chlamydia testing kit went on sale.

### Audit of the proportion of kits offered to EHC clients

As described in the Methods section, audit data on the number of EHC prescriptions by pharmacy, and hence the number of eligible chlamydia screening clients, was available for a total of 131 pharmacy months (cf Methods, Analysis 1). During those months covered by the audit 2718 women were given EHC but only 675 (24.8%) were offered a chlamydia kit. Although under-16s were most likely to have been offered a kit, there was no significant trend with age (Table 1, logistic regression trend test, p = 0.13).

**Table 1:** Age distribution of chlamydia screening kits offered to eligible EHC clients

	ALL	Under 16	16–19	20–24
Eligible	2718 *	205	1296	1217
Kits offered	675	65	321	289
%	24.8	31.7	24.8	23.7

* 4 eligible participants had missing age data

### Kit acceptance and return rates estimated from pharmacy tracking forms

Over the period of the study, pharmacists recorded offering 2904 kits (cf. Methods, Analysis 2), which were accepted by 1348 (46.4%) of women (Table 1). The acceptance rate differed significantly by age group (Table 2) with older clients more likely to accept the offer of a kit (p = 0.0005).

**Table 2:** Proportion of kits offered to EHC clients (based on pharmacy reports) and returned to the laboratory for *C. trachomatis *testing

	ALL	Under 16	16–19	20–24	p-value ^#^
Kits offered	2904	374	1197	1333	
Kits accepted	1348 (46.4%)	116 (31.0%)	566 (47.3)	666 (50.0%)	0.0005
Total kits returned^+^	236/1341 (17.6%)	23/116 (19.8%)	93/562 (16.5%)	120/663 (18.1%)	0.95

^+ ^7 kits were improperly recorded and were untraceable

^# ^Trend test for age using logistic regression

Only 236 (17.6%) of women who accepted a kit eventually returned a urine sample to the laboratory for testing (cf Methods, Analysis 3). Additionally, on the form returned with the kit, 13 gave their age as over 24 years, which would have made them ineligible for the scheme. There were no differences in return rates by age group.

### Number of cases of C. trachomatis among EHC clients who returned urine samples

Six samples were inhibitory and four leaked. Clients were re-contacted and seven repeat samples obtained and retested (one positive), giving 264 test results. In total, 24/264 (9.1%) samples were positive and the number of positives increased with age (Table 3, Trend test, p = 0.031). A logistic regression using actual, rather than grouped, ages showed a significant increase in positive tests with age (OR = 1.2 per year, 95% CI: 1.04 to 1.44; p = 0.015).

**Table 3:** Numbers testing positive for *C. trachomatis *by age group

	ALL	Under 16	16–19	20–24
Number of test results	264	20	119	125
Number Positive (%)	24 (9.1%)	1 (5%)	6 (5.0%)	17 (13.6%)
95% CI*	6.2 to 13.2%	0.3 to 23.6%	2.3 to 10.6%	8.7 to 20.7%

*CI: Confidence intervals

## Discussion

Women requesting EHC who have had unprotected sex, represent a discrete target group for chlamydia screening. In this operational study, designed to determine the uptake of screening in routine practice, only 25% of eligible clients were offered a postal kit. Older women were significantly more likely to accept the offer of a screening kit and had a higher risk of testing positive but were offered the lowest number of kits. The main rationale for making screening a priority in pharmacies is that it would be offered if the EHC request had been made in a clinical setting. Women who have accessed EHC in pharmacies may not refer themselves for testing.

The success of opportunistic screening depends on the assiduity of the health provider [7]. A strength of this study is that we have been able to demonstrate that pharmacists, though willing to provide EHC, did not consistently offer chlamydia screening. We tracked protocol deviations through the coding system so could quantify and allow for incomplete data recording by pharmacists. The EHC audit data provided an independent assessment of the proportion of missed opportunities. The audit did not cover the whole study period, but there is no reason to suspect that the periods covered were not representative. The audit indicated that pharmacists were not completing tracking forms for EHC clients who had not been offered chlamydia screening. Other postal screening studies that offered kits, either exclusively or partly through pharmacies, have relied on the numbers of kits distributed and returned as performance indicators [24,25] and did not assess the extent of missed screening opportunities. It should be acknowledged, however, that pharmacists may have been more assiduous in offering screening had they entered into a formal agreement with the local NCSP programme, or had they been offered additional financial incentives. This study predated the NSCP and did not include measures to raise awareness of the screening and its availability. It is possible that with greater awareness amongst women that uptake would be higher. Nevertheless, the Boots study in London, which took place after the NCSP started, also found that pharmacists were not pro-active in offering screening but waited to be asked for a kit by clients [11]. Selectivity or forgetfulness is avoided in schemes offering systematic screening – when a target population, defined by age, is contacted at defined intervals [26,27]. Consistency is more difficult to measure and address in busy commercial settings such as pharmacies, especially those with a high turn-over of pharmacists. In our study most pharmacies had only one EHC-accredited pharmacist and some of these left, causing both EHC provision and screening to halt.

Approximately a half of EHC clients accepted a kit but only 17% returned a sample. Some women may have felt obliged to take the kit without necessarily perceiving themselves to be at risk [28] or they may not have wished to consider their risk of chlamydia in the context of an EHC visit. For ethical reasons, it is difficult to find out why women refuse screening. Two studies reported that one of the main reasons that EHC clients refused chlamydia screening was recent prior testing [15,22]. The higher testing rate (over 50%) in the Department of Health Boots study may have been because more women actively requested screening kits [11]. They may have perceived themselves to be at risk or had symptoms, and so were more motivated to return a sample. We do not know why women take kits but do not return them but it is likely to reflect the quality of counselling provided, women's understanding of the purpose and importance of testing and their sense of obligation [29]. On-site testing would eliminate the waste associated with postal kits, and might encourage women for whom convenience of testing is important. It would, however, limit the range of pharmacies that could offer screening to those with facilities for urine collection.

The positivity rate for chlamydia was 9% but among Dutch 15–24 year olds who accepted a postal kit after collecting contraceptives at a pharmacy in a high risk-setting, 14% tested positive [25]. It is not always easy to identify high-risk settings, but selecting pharmacies to deliver chlamydia testing might be more productive, especially if the pharmacists were offered enhanced training. A review of educational strategies targeting primary care providers with a view to improving chlamydia screening rates indicated that the method and intensity of training is important [7]. In exit interviews 20/26 pharmacists admitted selectively offering screening to EHC clients who they thought would be more likely to have chlamydia. They mainly based this risk assessment on whether or not they perceived the client to be in a stable relationship. Their attitudes will be reported in detail in a separate qualitative study report. Pharmacists' selection of clients to be offered kits undermines the principles of an opportunistic screening programme where the onus is on the health professional to be actively case-finding in a population that may be unaware of the risk of infection [30].

## Conclusion

Targeted chlamydia screening represents a logical addition to the EHC protocol in pharmacies offering this service. Failure to offer screening to *all *EHC clients represents a missed opportunity for treating a well-defined high-risk group.

## Competing interests

The authors declare that they have no competing interests.

## Authors' contributions

LB conceived and designed the study and wrote the paper; GT collected the data and helped to write the paper; MH advised on, and conducted the laboratory tests; KB helped design the study, trained pharmacists and provided audit data; SR designed the study, undertook the statistical analysis and helped write the paper. All authors read and approved the manuscript.

## Pre-publication history

The pre-publication history for this paper can be accessed here:


